# Social Norms and Family Child Labor: A Systematic Literature Review

**DOI:** 10.3390/ijerph19074082

**Published:** 2022-03-30

**Authors:** Alhassan Abdullah, Inès Huynh, Clifton R. Emery, Lucy P. Jordan

**Affiliations:** 1 Department of Social Work and Social Administration, The University of Hong Kong, Hong Kong; aalhassa@connect.hku.hk (A.A.); ihuynh@hku.hk (I.H.); cemery@hku.hk (C.R.E.); 2 Department of Sociology, Haverford College, Haverford, PA 19041, USA

**Keywords:** child labor, social norms, culture, child rights, child work, family child labor

## Abstract

Background. Research has established the family as the predominant context for child labor practices. Decisions to involve children in child labor within the family or by a family member (herein family child labor) is strongly motivated by cultural beliefs that normalize child labor. This systematic review sought to synthesize evidence on the social norms that support child labor practices, and the normative interpretation of international child labor legislation/standards. Methods. We followed the PRISMA procedure for systematic review by reviewing empirical articles published between 2000 to 2021 and contained within the four key databases: Scopus, ISI Web of Sciences, PubMed and Embase. Findings from 13 articles that met the inclusion criteria were analyzed thematically. Results. The review included studies from three continents: Africa, Asia and Europe. Gender norms, informal apprenticeship norm, norms on succession and sustenance as well as obedience, were key social norms that influenced child labor practices in the family. Parents’ decision to involve children in child labor was strongly influenced by the collective acceptance of some occupations (e.g., cocoa farming and fishing) as family occupations, which need to be preserved, undertaken and passed on to children. Child rights and the UNCRC principle of children’s participation were considered foreign to most non-western countries and interpreted as contravention to the cherished social norm of obedience. The findings underlie the link between social norms and the common social values of resilience, hard work, and respect. Conclusion. The results provide foundations and target to develop normative change intervention programs to re-orient the negative interpretations of common social values and provide alternative pathways that prevent child labor within the social context.

## 1. Introduction

Child labor remains a major issue concerning child protection, despite increased research attention on its severity, causes and the intervention measures to curb it [1,2,3,4,5]. According to estimates by the International Labor Organization (ILO), over 246 million children (between 5 and 17 years) are involved in child labor, with 48 million located in sub-Saharan Africa [6]. Over two thirds of child labor cases happen in the agricultural sector, with more than 70% happening within the family unit [6]. Agriculture and other works undertaken by children in the family are “often hazardous in [their] nature and in the circumstances in which [they are] carried out” [6]. The fact that the family unit is regarded as a suitable context for child labor, where family members influence the involvement of children in labor, makes child labor within the family (henceforth family child labor) an important area and target for research into child labor. Literature on the causes of child labor underscore two critical antecedent factors: (1) economic forces and (2) cultural norms/values that support child labor [1,3,7,8]. Growing evidence has shown that the impacts of economic forces on child labor is mediated by cultural norms [9,10,11], and the cultural norms influence the choice families make for their children [9]. Even families who are stable economically have been found to allow their children to engage in work, e.g., using them as headsmen [12], highlighting the instrumental role of social norms in influencing child labor decisions within the family unit. Norms are deep-rooted beliefs that influence social actions within an institutionalized group. However, no study has so far systematically analyzed the sort of cultural norms and values that influence child labor decisions within the family. Social norms are predicted to have consequences on the health of people [13], mediated through the effects on health-seeking behavior, alcohol use and other risk factors [14,15].

Sociologists typically study social norms and their impact on social actions/decisions under the rubric of Parsons’ statement; legitimacy of social values are unquestionable, however normative interpretation of the values differ and often matters the most [16]. Much research on the impact of cultural values and social norms follows Parsons’ [16] argument by seeking to examine the different pathways social values are interpreted to influence social actions and decisions. Primary research on the influence of cultural values on child labor has shown that parents’ decisions to involve children in child labor are influenced by their desire to ensure that children secure a better future [17], become hard working people [18], are resilient to life challenges [19] and to ensure their adequate development [18]. Inherent in these justifications of child labor are the normative interpretations of common social values, such as hard work, success, and resilience, within the culture. They show that normative interpretations matter in efforts to change values in communities. On the other hand, the value of success in other contexts may not be normatively interpreted with associated social actions that bear on involving children in family work/child labor. Instead, they may be operationalized through enrollment in education. For instance, Green [20] reports that Western perspectives on childhood highlight the need for children to be free from adult responsibilities and acquire the best formal education to ensure their success in the future. Varied normative interpretations of common social values and their influence on family child labor decisions led us to argue that social norms play a central role in an attempt to understand and address the child labor phenomenon. Weiner [21] reports cultural values among the key antecedents of children’s work. This systematic review of existing literature seeks to examine the social norms that underpin child labor within the family in order to provide systematic evidence to inform research and intervention programs.

### 1.1. Social Norms, Child Work and Family Child Labor

Child labor in the family is a contested concept, especially in the context of cultures that traditionally require children to perform domestic duties and support their parents as part of their childhood training [22,23]. Whilst some argue that child work is an essential requirement for the socialization of children [24,25], others have found child work as an architect to child labor, child exploitation [26,27], and modern slavery [28]. The International Labor Organization (ILO) defines child work as all productive activities that are carried out by children (either in a family business or common market; remunerated or not) that last for a minimum of 1 h duration within a day [6,29]. Duration of work, nature, intensity of work, and the age of the child are typical features that determine child work. Similar features determine the judgement of child labor [30]. The ILO Convention 138 [31] sets a minimum threshold of 15 years for a child’s admission to work, and 18 years for hazardous work [6,32]; but many developing countries have the flexibility to admit children aged 14 years to engage in “light work”. So far, the extant literature on child work and child labor have agreed that there is a very thin line between the two concepts (child work and child labor), which is often difficult to determine until they have happened [29]. They are difficult to differentiate in practice [29], and vary from nation to nation [33]. It is more difficult to differentiate child work and child labor in localities that have sanctioned child work as part of the socio-cultural traditions [6]. White [27] highlights the complexities involved in the two concepts.

*“It is impossible to draw a clear and unambiguous line between ‘child work’ (the more acceptable forms of children’s work, which are relatively unharmful and in cases may even be beneficial) and ‘child labour’, the unacceptable, exploitative and harmful forms of children’s work, a ‘social evil’. Most attempts to draw such a line are either too general, vague and circular to be of use, or if they try to be concrete and specific, are too contradictory and illogical, and out of line with the views of children themselves.”*

[27] (p. 837)

Essentially, the argument suggests that promoting child work may risk promoting child labor within the family unit. This is particularly the case as recent child labor statistics identify the family unit as the main contexts where child labor thrives [6]. Nonetheless, evidence on the central role of culture in underpinning child work and child labor suggests that culture or social norms could be an important source in efforts to understand how child work and child labor can be differentiated, understood and prevented.

Similarities in the social norms and value aspirations that underpin both child work and child labor also convolute the seemingly cybernetic relationship between child work and child labor in most cultural contexts. Children are considered to work on family farms as part of their preparation for adulthood, and to acquire basic skills that are integral for their development [18,34]. Children’s involvement in family farms increases family income and enhances family standard of living [17]. Their participation in fishing and family cocoa farms are regarded as important means to secure better future for children [17] and to maintain family businesses [35]. Similarly, it is believed that the involvement of children in labor increases their affection for the labor market and enhances their personal development [19]. Engaging in child labor is considered an acceptable pathway to prepare children to be hardworking adults, and resilient to future work challenges [19]. Children in Turkey are found to work during their early years in order to contribute to family farm work [36]. Inherent in these justifications of child labor and child work are normative interpretations of the values of hard work, resilience, success, and achievement. Furthermore, decisions and attitudes towards child labor in Africa are also informed by the communal expectations of respect and mutual interdependence [37].

Social sanctions, including stigma against parents, compel them to conform to the norms that support child labor practices [38]. Lopez-Calva [39] theorized that there is a direct relationship between social stigma and child labor. Stigma against parents who do not support child labor norms (fail to involve their children in child labor) is found to be associated with increased child labor practices. The desire to obtain social acceptance, avoid the likely cost of stigma and social rejection, increases the utility of engaging children in child labor. The critical role of social norms in child labor practices led us to systematically examine specific social norms in the literature and unravel ways they inform family child labor practices. Such an advancement is critical to inform further research and the development of concrete normative campaigns that shift social norms in communities.

### 1.2. Social Norms and Legislation on Child Labor

Local social norms that support the involvement of children in work and child labor may likely be in conflict with international standards that frown on child labor practices. Communities are more likely to value their local norms that favor child labor and to to discredit international child labor legislation. Legislation and standards to regulate child work and prevent child labor have been in existence since the identification of child labor as a major social problem affecting the growth, health and wellbeing of children [40,41]. Led by international organizations (such as ILO and IOM), international promulgations on standards to prevent child labor have been developed and revised consistently to protect children. The United Nations Convention on the Rights of the Child (UNCRC) [42], the ILO Convention No. 182 on the Worst Forms of Child Labor (1999) [43] and the ILO Convention No. 138 on the Minimum Age of Employment (1973) [31,44,45] are the key international legal frameworks that regulate child labor practices. Article 32 and 35 of the UNCRC spell out issues related to the abolishment of child labor, forced labor and the need to protect children from all kinds of work exploitation. Principles in the UNCRC are developed based on the western conceptualization of childhood [46]. However, some of the commitments are echoed in other regional legislation such as The African Charter on the Rights and Welfare of the Child [47], and operationalized in the local laws of most countries. Overall, the UNCRC has an overriding commitment and target for member countries to ensure that children participate in decisions that affect them, are protected from harm, and enjoy the rights to life, survival and optimal wellbeing. However, the implementation of this legislation and its impact on children are largely influenced by social norms and values [48]. The rights of children in some contexts (e.g., South Africa) are seen as part of measures to undermine the authority of parents, traditional leaders [49] and elders [50]. Children in Africa are expected to act in response to the wishes and demands of their parents [51,52,53,54]. Children’s participation in cocoa farms is normatively interpreted as a form of agency [55]. However, the standard participation procedure spelled out in the UNCRC that allows children to exercise autonomy is considered an act that spoils children [55]. The foregoing evidence highlights the various international child labor legislation that could be interpreted within the context of local social norms, and how that influences family child labor practices. Therefore, additionally, the aim of this systematic review is to identify the various ways international child labor standards are interpreted in local contexts by seeking responses to the following key questions:

**Guiding Review Questions**

- What social norms precipitate child labor practices?
- How are international child labor legislation/standards normatively interpreted in the context of child labor?

## 2. Method

Guided by the Preferred Reporting Items for Systematic Reviews and Meta-Analysis (PRISMA) [56,57] and recommendations by Rew [58], we conducted a comprehensive literature search to synthesize evidence on social norms that influence family child labor practice. The use of the PRISMA guideline provided a transparent framework to synthesize data following scientific principles that enable replication. Rew’s [58] suggestion helped to make vital decisions in the review process, including selecting relevant databases, setting important criteria for including studies and criteria for the screening and assessment of the quality of included studies.

### 2.1. Search Strategy

Four academic databases including: Scopus, ISI Web of Sciences, Pubmed and Embase, were searched. The literature searches were conducted from June to December in 2021. A combination of key words on child labor and social norms, together with Boolean operators were used in the search. Table 1 presents the keyword combinations and Boolean operators used in the search. York dare database, Cochrane library and PROSPERO database were searched to confirm the review has not been replicated elsewhere. Snowball search and expert recommendations supplemented the search procedure.

### 2.2. Inclusion Criteria

Following the purpose of the systematic review, a clearly defined criteria was developed to guide the screening of studies. Studies that satisfied the following criteria were included:

- Articles reported qualitative or quantitative evidence (or both) on cultural norms that influence child labor practices. Additionally, (or) reported evidence on the normative interpretations of international legislation that seeks to prevent child labor practices.
- Articles reported findings from the views of any of the following groups (or a combination of any of them): community members, children, parents and social service workers.
- Articles are empirical studies published in English, between 2000 and October 2021. The benchmark of 2000 was chosen in line with the promulgation of the ILO standards on Worst Forms of Child Labor (1999), (*n*.182), ensure that the findings are relevant to current legislation and guidelines on child labor.

### 2.3. Search Outcomes and Screening

**Figure 1:**
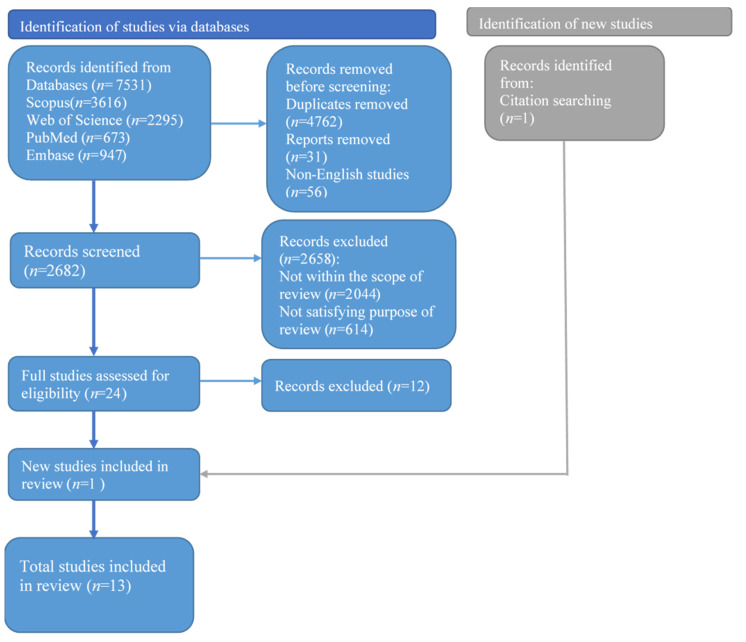
PRISMA flow diagram.

A total of 7531 results were obtained from a general search through the four databases. Title, author names and other reference details of the studies were exported and saved into an excel spreadsheet. This allowed for duplicates to be automatically searched using the duplicate function in excel. In total, 2682 results were retained after duplicate titles were searched and removed. Articles that were published before 2000 and published in languages other than the English language (*n* = 56) were removed before screening. The remaining articles were screened. Article titles that were beyond the scope and purpose of the study were removed (*n* = 2044). This included book chapters and non-empirical studies. Six hundred and fourteen (614) articles were deemed as not satisfying the purpose and criteria for inclusion in the study. The remaining 24 articles were read thoroughly and 12 were removed as their discussion of social norms were not in the context of child labor. One additional study was found after a snowball search through the references of the 12 articles. The entire search and screening procedure was conducted by the third author and validated by the first author. The researchers conducted weekly meetings to discuss the search and screening process. There were no major disagreements with respect to judgements on the eligibility of studies. Minor disagreements, which mostly happened in the quality appraisal of studies, were keenly discussed with the senior authors (author 2 and 4). The screening process is presented in the PRISMA flow chart shown in Figure 1 below:

### 2.4. Quality Appraisal

We assessed the quality of the included articles using the quality appraisal tool by Salzmann-Erikson and Dahlén [59]. A 25-item checklist was used to assess the quality of empirical studies, mostly qualitative studies, by focusing on the methodological rigor of the articles. Key questions on the appraisal checklist included: “Are the criteria for inclusion described?”, “Does the study’s title correspond with its content?”, “Are the criteria for exclusion described?”, “Is the study’s purpose clearly answered?”, “Is the data systematically collected?”. Each item was assessed using a dichotomous response of “Yes” and “No”. Articles are rated as low quality if not more than 17 items of the quality assessment checklist are rated “Yes”. Articles with 18 to 20 points “Yes” ratings are considered to be of medium quality, whilst those with more than 20 points “Yes” ratings are considered to be of high quality. Six of the included articles were considered low, two medium and five as high quality. The ratings give a general idea of key information that were missing in the published articles, but they were not used as a basis to remove articles from the study. The appraisal was performed by the first author and validated by the third author. No significant difference was recorded in the assessment by the two authors.

### 2.5. Analysis Procedure

We followed Braun and Clarke’s [60] recommendation for conducting reflexive thematic analysis in analyzing evidence from the included articles. As part of the screening process, each article was read thoroughly, and notes on key ideas and findings were written in the comment section of Adobe PDF reader program. The written notes captured key findings, overall ideas of the study and key contributions of the study. Direct quotes from participants in most of the articles were linked to the notes. The quotes, key ideas and author’s written notes were organized in excel for further analysis. The written notes were again reflected upon in line with the meanings they connote and their context. The written notes were then refined and stated as codes. Examples of codes included “decisions undermine respect” “part of culture to work” “we have to contribute to family income” “children are cheap labor” and “cocoa and fishing are family work”. A list of all codes from the 13 articles were organized in word together with a short description of the meaning and nuances. This enabled the researchers to compare the codes and merge them to form themes under the rubric of social norms. The core themes included: gender norms, obedience, and informal apprenticeship norm. Codes were merged based on their similarities in terms of the interpretations of particular social norms or values. A detailed report of the themes is discussed in the results section.

## 3. Results

### 3.1. Study Characteristics

Out of a total of 7531 articles and reports retrieved from the broad search, 13 articles met the inclusion criteria. Four of the included articles by Adonteng-Kissi [61,62,63,64] were published from two broad studies. Hence, the total included articles emerged from 11 unique studies. Broadly all 13 articles included in this review examined the social norms in child labor under the rubric of understanding the causes of child labor, particularly the social and cultural causes. All articles (except [65]) involved interviews with children and parents [61,62,63,64,66,67,68,69,70,71,72,73], including children who are victims of child labor and parents who are perpetrators [61,62,63,64]. Six of the articles included the views of social services workers, and workers of Non-Governmental Organizations (NGOs) [61,62,63,64,65,67]. Most of the studies used a multi-method combination of qualitative in-depth face-to-face interviews, focus group interviews, ethnography, participatory research and/or observations [61,62,63,64,65,66,68,70], whilst the remaining studies used single methods: in-depth interviews [67,72], ethnography [69] and a combination of in-depth interviews with surveys [71,73]. The majority of the studies were conducted in sub-Saharan Africa (*n* = 10) [61,62,63,64,66,67,68,69,70,71,72,73], two in South Asian countries; Nepal and Bangladesh [69,72], one in Central Asia (Tajikistan) [67], and one in South Eastern Europe (Turkey) [68]. Child labor within specific occupations such as cocoa [65,70], carpet industry [69], fishing and farming [61,62,63,64,66] and domestic work [71] were the focus of most studies. However, 3 of the 13 articles focused on a general description of child labor and child work [68,72,73] without specifying a particular occupation. Even in such studies, participants linked their responses to child work or child labor in domestic work, farms and factories. A summary of the included studies is provided in Table 2.

**Themes related to social norms precipitating child labor**

### 3.2. Informal Apprenticeship Norm

A common finding from most of the articles (*n* = 8) is that children are culturally expected to receive informal apprenticeship training in the family by engaging in their parents’ primary occupations. The rationale behind the social norm of child informal apprenticeship is supported by narratives from key stakeholders, including parents [61,62,64,65,68,71], child victims of labor [67] and agricultural coordinators [65]. Parents in a Ghanaian study [61] argued that the informal apprenticeship for children is needed to prepare children to be tough so as to meet future life challenges. A parent described how this justification influenced her involvement in street vending during her childhood:

*“In my own situation, I used to be a newspaper vendor on the streets. I had to sell newspapers on the street of Accra in the morning before going to school. My parents used to tell me that they were training me to be tough in life so that I’ll be capable of facing the challenges of life.”*

[61] (p. 474)

Children are believed to become hardworking when they start work early. This cultural notion underlies Turkish parents’ decisions to involve their children in work.

*“I want them to start working now, so that they get used to working and not become loose.”*

[68] (p. 46)

The rationale for training children to be tough, and hardworking through child work and child labor is complemented by the cultural belief that engaging children in labor secures a better future for them. It is the shared normative belief that children’s participation in work at early ages imbibes in them a natural spirit of hard work and makes them resilient to work-related challenges in their adulthood [62,65,68]. A parent, who is identified to have involved her child in cocoa farming, emphasized the need to engage children in work in order to ensure they have a better future.

*“Our culture teaches our children to believe that work socialization is meant to provide training which will ensure a better life in the future.”*

[61] (p. 472)

The early child apprenticeship training is conceived normatively to inculcate strong work ethics in children, leading them to become hard workers, resilient and ultimately to achieve success. Evidence from parents in Ghana [64], Turkey [68], and Côte D’Ivoire [65] confirmed the cultural justification that children learn better [65,68] and obtain strong work ethics [64] when they start work early. Narratives from some parents confirmed that these cultural justifications influenced their decision to involve their children in work, albeit arguing that such work does not constitute acts of child labor.

*“Whatever work I assign to my children is not intended to harm them but to train them to have strong work ethics that will in turn help them to have a better future.”*

[64] (p. 59)

The narrative confirms that the interpretation of social norms does indeed matter in influencing social action [16].

Additionally, inherent within the normative justification of securing a better future for children through child labor and child work is the notion that work enhances children’s level of creativity [63]. It is also meant to increase and sustain their interest in work (confirmed by evidence from Ghana, Tajikistan, South Africa and Côte-D’Ivoire) [64,65,67,71]. Akilova’s [67] study among children working in Tajikistan revealed that children have normalized these interpretations and as a result they consider work as a normal part of childhood. However, it appears that the children have been conscientized to consider work as part of the training within the family. As a result, they do not consider work in or with a family member as a classic form of “work”. A twelve-year-old child who started working at age 2 narrated her experience this way:

*“I actually started working at age two–not really working though. I went with my cousin on herbike to sell refreshing drinks and tea to people in the market. I just accompanied her. I started working on my own when I was 12.”*

[67] (p. 240)

The notion of child work as a normal part of childhood was found to be stronger in rural Tajikistan [67], similar to the evidence from Ghana [64]. The legacy of the Tajikistan community, which focuses on developing children to become productive members of society, may have enforced the norm of child labor and child work, even among children [67]. Particularly, it was found that children between the ages of 12–14 were considered matured in Tajikistan.

When quizzed on how they considered child work within the family, some Ghanaian parents justified that working in the family is a traditional form of schooling, which provides alternative sources of livelihood for children [64].

*“Engaging my children in fishing is simply a traditional system of schooling which doesn’t violate the rights of my children.”*

[64] (p. 60)

However, parent’s view on child work and child labor as alternative sources of livelihood for children appears to be strongly influenced by the high rate of graduate unemployment. The high rate of graduate unemployment made formal education unappealing to parents, which enforced their beliefs in the norms of informal apprenticeship. A parent in a rural community in Ghana affirmed this notion in her narrative:

*“Why should I enrol my children in school if they’re going to be unemployed after finishing? For me, I believe it will make more sense to engage them in my farming for them to acquire some skills that will help them in their future lives.”*

*“Why should I enrol my children in school if they’re going to be unemployed after finishing? For me, I believe it will make more sense to engage them in my farming for them to acquire some skills that will help them in their future lives.”*

[64] (p. 59)

The evidence highlights the strength of the social norm of informal apprenticeship in the Ghanaian society.

### 3.3. Gender Norm

Diverse interpretations of gender roles and expectations appeared in most of the studies as antecedents to child labor. Societal expectations on the role of males and females influenced child labor practices, and the nature of child labor related activity children are engaged in. Men are trained to become breadwinners in the family, with women as family maintenance agents (*cf*. [62,64,65,67,72]). As a result, child labor in income-generating activities, such as fishing, carpet work and cocoa, was found to be common among boys, whilst girls predominantly engaged in domestic duties [62,64,72]. Most female parents confirmed the decision to engage their young girls in domestic chores as part of their training to become good wives.

*“I want to use my personal experience as an example. I was the only girl amongst seven boys, so I needed to be familiar with the demands of marriage life as is required of all girls. When I was living with my siblings, I used to wash, clean and sweep for them because I was being prepared for future life. The role of a girl in the household is to provide services to the boys.”*

[62] (p. 13)

The cultural belief that a good wife is one who satisfies her husband’s family’s expectations influenced parents in Tajikistan to engage their children in domestic chores at their early ages [67]. Similarly, young boys in South Africa engaged in hard labor to feed their family as part of their preparation to become breadwinners for their future families [71]. It is reported that the gendered notion on child labor is enforced by social sanctions. Boys in Bangladesh who undertake household chores are labelled as “women” and called *names,* and vice versa [72]. Their parents are also seen as bad parents.

*“If men do these jobs [housework] everyone will call him a woman.”*

[72] (p. 13)

It is the cultural expectation that children carry out the culturally defined occupations/activities which prepares them adequately to assume their roles as breadwinners and family maintenance agents.

### 3.4. Asset Value of Children

Themes and narratives from the included studies revealed that societal standards that support the asset value of children also influence child labor in the family [62,63,64,67,71]. These social standards underlie the economic importance of children’s contribution to family work and family income. Parents are therefore encouraged to engage their children in their own occupations so as to benefit from their economic value. Children are more likely to be involved in child labor in families where parents’ occupations demand a lot of labor.

*“Fishing is a very difficult task and parents need more labour to be able to carry out the task. In my view, government should allow children to support their parents and supplement household income. At the low income we get from our fishing business, how can we hire only adult labourers?”*

[62] (p. 10)

The narrative shows that children are cheap sources of labor, as well as alternative sources of labor. The notion of children contributing to families is endorsed by parents in South Africa [71], Ghana [63,64,65] and children in Tajikistan [67]. According to parents in Ghana and South Africa, certain kinds of family occupations, such as fishing, farming and family business, require *more hands* [more labor] with specific tasks assigned for children in the family [63,64,65,71].

*“This is because there is inadequate mechanisation of artisanal fishing industry. The work is quite difficult, and fishermen need more hands.”*

[64] (p. 60)

The need for more hands due to the lack of mechanization of fishing (use of modern fishing methods) influenced parents to involve their children in child labor. This was confirmed by another parent:

*“I think many parents are using their children to work because their work is not mechanised. The State needs to support parents to mechanise their work to help eliminate child labour.”*

[64] (p. 60)

A chronological analysis of the above two quotes confirm that children are indeed engaged in hard work as they are meant to work in non-mechanized family fishing practices. Promoting mechanized fishing practices may provide leeway to avoid hard work in family fishing but may not completely remove children from working in family fishing.

The social expectations for children to contribute to family income is accepted by children as part of their mandatory responsibilities to their family. Some children in Tajikistan revealed that it is their obligation to contribute to their parents’ work/business so that their parents could save money (meant for hiring other staff) [67]. Children feel enthusiastic when they judge themselves to have satisfied this societal demand [64].

### 3.5. Value of Formal Education

A normative interpretation of the value of formal education was found to inform parents’ decisions to involve their children in child labor. Albeit diverse, there was an argument about the relevance of formal education, which influenced parents’ decisions to engage their children in labor [64,67,68,69]. Parents believed that informal apprenticeship in family work provides future job security for children, compared to formal education. Due to the low job security arising from formal education, children are believed to be better off receiving an informal apprenticeship from their parents’ occupations. The relative importance of informal apprenticeship training over formal education influenced child labor practices.

*“Parents tell us that they know children in the community who have successfully completed their education, yet they don’t have jobs. Some parents believe putting their children into farm work is worthwhile because their children develop their employment skills that get them ready jobs.”*

[64] (p. 60)

In the context of poor school quality, low job security after school and severe challenges with school access, parents may rationally prioritize informal apprenticeship through child labor over enrolling their children in school. The high rate of graduate unemployment in many developing countries was found to support the shared belief that informally training children in family work is a better option as opposed to formal education (*cf.* [64,65,66,68]). Some parents are further encouraged by the cultural belief that “education does not fit every child”. Parents who held this belief suggested that children who are stubborn do not require formal education, instead they should work in family farms [64].

On the other hand, some parents justified their decisions to involve children in child labor as part of the measures for children to appreciate the value of formal education. They argued that education is without stress since it does not involve hard work. Therefore, children’s experiences of both hard work and formal education would enable them to assess the two occupations and appreciate the value of formal education (see [68,69]). The following narrations from parents in Turkey exemplify their argument:

*“I swear to God, I’m not after their money…. They don’t work and they don’t care about school. I’m saying if they work, maybe they will understand the value of school and go to school.”*

[68] (p. 46)

*“I say maybe if they work, going to school will feel sweeter [better] to them. For instance, because work will be too hard, school will be [easier].”*

[68] (p. 46)

Even though the overall intention for engaging the children in hard work (child labor) is to make them accept formal education, the rationale can be defeated when the children develop a liking for the work compared to school. That said, evidence from children in Nepal suggests that children who experienced the two; formal education and family child labor, found education to be the better option [69].

### 3.6. Sustenance and Succession of Family Occupation

A considerable number of the studies reported findings on the social norm of sustenance and succession of family occupation [62,64,65,67,73]. Works undertaken by parents, such as farming and fishing, are conceptualized as family occupations which are supposed to be assumed by their children and sustained for future generations [64]. Evidence from parents, social service workers and NGO workers in Ghana confirmed that cocoa farming and fishing are classical family occupations which children should strive to maintain. Family occupations give identity to the family. The desire to maintain family occupation enforced parents’ commitment to involve their children in cocoa farming and fishing [64,65].

*“Most parents are farmers in these rural communities, and very often they want their children to help them on the farms because they want to socialise their children to take over from them. However, this cultural practice is sometimes abused by some parents. Some parents engage their children in work in times that those children are supposed to be school. Children support their parents on the farms to perform tasks such as weeding, planting of seedling and burning unwanted woods in the farm. Many boys work for 8 h on the average in a day.”*

[62] (p. 9)

Commitment to the norm of sustenance and succession of family occupation made parents opposed to the idea and efforts to eliminate child labor (see [62,64,65]). Even professionals, such as NGO workers, who are mandated to ensure the application of child labor standards and implement measures to eliminate child labor, are sometimes influenced by these social norms.

*“I have been in this community long enough to know that many farmers want their children to be socialised and take over from them and therefore they won’t agree to total elimination of child labour.”*

[62] (p. 10)

The findings highlight ways deep rooted social norms can influence child labor and obliterate the commitment to eliminate child labor. Training children on family occupations is considered the primary responsibilities of families to ensure that the next generation of farmers are produced [65]. A cooperative director for cocoa farmers in Côte D’Ivoire explained:

*“And also we have to prepare the future generation of farmers. So, when the child is in the farm, there are some activities that he can do and some other activities that they can’t. He must assist. He look at his father working. And then the child keep it in his mind. So the child, during his free time, his holidays, must go to farm and see what his father is doing.”*

[65] (p. 8)

Krauss’ [73] household survey among Ghanaian parents and key informants, such as the Minister of Education, showed that top level policy makers are also influenced by these social norms. An indication that social norms on child labor cut across various strata of society. A quote from the Minister of Education in Ghana showed ways the social norm of succession influences child labor:

*“…I belong to the old school, where you ‘bring up the child the way he should go’ so that if you are a child in a farming family you should be able to learn about farming from your parents…”*

[73] (p. 551)

The desire and commitment to uphold the norm of sustenance of family occupation is enforced by the notion that family occupations will collapse if children are not trained to assume and continue with it (*cf*. [64,65,73]).

*“Our family businesses will collapse if we take our children out of fishing. It is part of our culture for children to get deeply involved in what we (parents) do.”*

[62] (p. 11)

**Themes related to the normative interpretation of child labor standards**

Only 2 of the 13 articles reported evidence on the normative interpretations of child labor and related legislation or standards [61,66]. The finding discusses parents’ interpretations of the UNCRC sections on children’s active participation in decisions, and children’s rights. Evidence on the two themes is discussed below:

### 3.7. Obedience

Parents opposed the UNCRC commitment for children to be given the free will to make decisions and contribute to decisions that affect their lives [61,66]. Children’s rights to active participation in decisions were interpreted within the normative cultural framework of obedience and respect. Children’s active participation in decisions were considered among the potential practices that deviates from the cherished norm of obedience and respect of elders [61,66]. Children are culturally expected to obey the decisions of elders and take instructions from persons who are older than them, especially their parents. Thus, the power to make decisions within the family rest upon elders, who also have the cultural authority to make decisions for children [61,66].

*“Ghanaian culture gives parents more power over their children. Thus, there is virtually no children’s rights to participation in family decisions since parents’ views on matters concerning the child carries a lot of weight. It is quite difficult for children to disobey their parents in matters relating to child labour.”*

[61] (p. 472)

Legitimacy of parents’ decision-making power and the social expectation of obedience influenced children’s acceptance and commitment to instructions from their parents [66]. Those who obey and submit to parent’s instructions, without questioning, are considered to be good-natured [64,66] and respectful children [71]. The incentives associated with being considered a well-natured child further strengthened children’s commitment to the belief that adults are better positioned to make decisions for children [71]. Additionally, the cost associated with being branded as a bad parent [61], and having your children considered as “wayward” [64] influenced parents’ conformity to the norm.

### 3.8. Child Rights in Child Labor

Broadly, Adonteng-Kissi’s [61] study explored parents’ and stakeholders’ opinion on children’s rights within the context of child labor in the Ghanaian cultural context. Collectively, parents agreed that a child’s right is a foreign concept that contravenes the social norms in Ghana, particularly the social norms that support child rearing practices. They argued that children’s rights spoil children and make parents powerless in their efforts to guide and nurture their children towards a successful future. They believe that engaging children in the works of their parents is not wrong, as that has been the practice since the inception of traditional communities. Evidence from the narratives of parents who involved their children in child labor revealed that government officials are reluctant to implement children’s rights and child labor related legislation, because they agree that child work is an old-aged practice that is good for children. Effective child support, interventions for struggling parents and strong government enforcement [61] were identified among the key resources and structures required to ensure the effective adoption and implementation of children’s rights to end child labor in Ghana. The lack of these important requirements makes children’s rights unfit for the local Ghanaian context. It was suggested that the social norm that spells out the need for children to work and complement family income may change if there are adequate social interventions to support child rearing and family wellbeing. The lack of such interventions makes child rights meaningless in the local context. Citing western countries, the parents believe that child rights have worked in such contexts due to the existence of strong social interventions and legal structures that ensure compliance.

## 4. Discussion

This study systematically analyzed evidence from existing studies on the influence of culture and social norms in child labor. It further analyzed evidence on the normative interpretations of legislation and international standards that seek to abolish child labor practices. The findings, which have been categorized under six specific themes, are discussed within the context of cultural values that underpin the social normative interpretations.

### 4.1. Social Norms Precipitating Child Labor

A common theme from the studies suggests that social norms on informal apprenticeship training for children influence child labor practices in many contexts, including Africa, Europe and Asia. Child labor practice is strengthened by the cultural belief that involving children in informal training, either through family occupation or household chores, imbibes in them the spirit of hard work, strong work ethics and prepares them adequately to face life changes. Johansen [17] reports that there is an overriding acceptance of the belief that child labor and child work prepares children adequately for the future and makes them hard workers. Similar to the findings by Verner and Blunch [19], it is evident that parents are more willing to engage their children in work early to develop affection for work and good work ethics. It is therefore not surprising that children in Tajikistan were reported to start work as early as 4 years. The findings collectively highlight societies’ commitment to the social values of resilience and hard work. Values of resilience and hard work are influential in society, and considered cardinal components of common values that needs to be promoted [74,75]. However, the normative interpretations of these values, is found to be undesirable for some children. If children can only become hard workers when they start work early, or when they engage in child labor, then the relevance of hard work should be questioned. In the same vein, if resilience can only be achieved through child labor, then the value of resilience should be given a second thought. The findings is indicative of societies’ commitment to social values, which are instrumental for group membership [75] and survival. It also demonstrates that normative interpretations of these values, in terms of practices to achieve the values, differ and in some cases could be detrimental to the health and wellbeing of children (especially in the case of child labor). Although engaging children in farm work could be hazardous, parents may be compelled to send their children to work to ensure proper supervision and care. This may happen when there is no proper supervision for children at home, or when children could be at higher risk of experiencing violence at home. These adverse consequences, resulting from leaving children at home, may explain parents’ views on sending their children to farm.

On the surface, the values of resilience and hard work could lead to uncritical acceptance from people because they ultimately lead to success and wellbeing. This uncritical acceptance was evident in this study as children in some studies accepted their parents’ decisions to involve them in work, and considered child labor as a normal part of their development [67,70]. Normalization of child labor by children shows the intergenerational transmissions link of social norms that enforce child labor practices. Thus, it is important for interventions to target at breaking this intergenerational link, through a wholistic normative change intervention.

Some parents involved in the studies alluded to the influential role of gender norms in child labor. Gender norms and role specification informed child labor practices. It emerged that the acceptance of males as breadwinners, and females’ primary role of maintaining the family informed child labor decisions. As a result, most boys were found to engage in income generating works, such as cocoa farming, fishing and work in industries, whilst females were engaged more in domestic chores. The study by Delap [72] in Bangladesh found that boys who engaged in household chores were labeled as “women”. This social stigma reinforces the commitment to the gender norms as it introduces informal social control mechanisms. Evidence on gender norms in child labor also suggest that, overtime, child labor would be gender specific; whereby boys will engaged in income generating labor, with girls involved in household maintenance labor. If this preposition should hold, in the long run hazardous child labor and non-child labor would assume similar dimension [76].

Parents in the studies provided narratives that underlie the social norms on the economic value of children. Parents from rural and urban Ghana, South Africa, Tajikistan and Turkey [62,63,64,67,71] expressed the belief that children are traditionally mandated to work and contribute to family income. It emerged that certain occupations, such as fishing and farming, required the collective involvement of family members including children. There are specific tasks for children in such occupations. Evidence from the cross-continental studies mainly demonstrate the normative interpretation of childhood, which zoom into the long-standing debate on childhood and child labor [77,78,79]. Indeed, conceiving children to have economic value transgress the global accepted standards on childhood (contained in the UNCRC), which are mainly based on the Western conceptualization of childhood. These are imposed western standards; however socioeconomic improvements are needed to change values. The fact that the normative standards on the economic values of children is also accepted by children suggest that a different conception of childhood may be at work in such context. Viruru [79] has argued that the interaction between childhood and child labor is complex and this complexity could be fueled by cultural factors. Findings from the included studies show that the normative interpretation of the economic value of children undermines efforts to attain the global standards on childhood.

Children were likely to engage in child labor in a context where family occupations are commonly practiced. This is in line with the cultural commitment to ensure sustenance and succession of family occupations. Common occupations identified with child labor, such as farming and fishing, are traditionally defined as family occupations, which are required to be sustained and transcended onto the next generations. Involving children in work is a common pathway to ensure the sustenance of family occupation.

### 4.2. Normative Interpretation of Child Labor Standards

This review shows that children’s right to participate in decisions that affect their lives are interpreted within the normative framework of obedience and respect. Studies mainly from Ghana [61,66] have shown that children are not accorded the freedom to exercise their right to participation because child participation is culturally interpreted as measures that spoil children. Child participation could lead to children disrespecting adults, since the Ghanaian culture gives enormous power to parents and adults and expects children to listen to instructions from adults [51,53]. Child participation could also undermine the authority of adults and elders in the community. It can be argued that the normative interpretation of child participation right is part of the conscious cultural efforts to achieve the value of respect and obedience to authorities and parents.

Finally, children’s rights in general were considered by parents as alien and part of the measure that spoils the African child. It is argued that children’s rights are geared towards making parents less powerful in efforts to guide their children. However, the study did not specify types of rights that are alien to the African context and those that spoil children [66]. It is generally accepted that rights that empower the child and seek to make them independent contravenes the African culture, which promotes obedience and respect of elders’ decisions and instructions [80,81,82]. Such rights are deemed to challenge parents’ authority in the family. Yet, the African Charter on the Rights of the Child provides measures to promote children’s rights to decisions, participation and protection. This suggests that children’s rights may not be entirely foreign in the African context. Narratives from the parents confirmed that child work is an accepted pathway for children to build responsibility and independence. This may imply that the length of children’s involvement in work could vary by the socioeconomic differences within the family and community. In low-income families, children are forced to grow up quicker by taking on work and responsibilities, compared to children in middle-high income families where adolescence can be prolonged into the 20′s, varying by context and cultural differences.

### 4.3. Implication for Child Labor Practice and Research

Evidence discussed in this review has some implications for practiced measures that are required to eradicate child labor. The findings show that varied normative interpretations of key social values: respect and obedience, hard work, resilience, and sustenance and succession, influence child labor decisions. These negative normative interpretations are deep-rooted and legitimatized among children. A multi-pronged normative intervention program that seeks to change the negative normative interpretations is needed. In particular, a combination of normative change campaign with community education is needed to intensify education on the negative social norms. The education should aim at sensitizing communities to acknowledge the unfavorable outcomes of the existing normative interpretations of the common values and provide alternative justification/avenues communities can model into their accepted social standards. Children should not be left out in the normative change interventions because they appear to have legitimatized the negative interpretations that support child labor practices. Social sanctions should be instituted to control negative social norms and ensure conformity to the newly developed social standards. Modelling new social standards could be challenging. As a result, we advocate for a wholistic community approach that involves three stages: identification of the negative social norms on child labor, collectively develop alternative normative paths, and collectively develop implementation plans including social sanctions to ensure social control. Other important strategies that can help eliminate child labor include measures to promote access to school, increase school supplies, reduce transportation costs for students, and a conditional cash transfer program to stay in school. The school, health services and governmental programs can play a role in promoting children and parents’ understanding about the hazards and benefits of family’s work. Such holistic approach, involving efforts from the educational system, government, health and economic system (conditional cash transfer) will be useful to change the values and norms. It will help children and their families to appreciate the realities regarding how they are interpreting values/social norms and the impact on children.

A periodic evaluation of the child labor elimination interventions is needed to ensure that the adverse consequences are identified and curtailed (*cf*. [83]). Child labor elimination programs may have hidden and undesired consequences of increasing child labor within poor communities [83]. These poor communities may develop alternative occupations/avenues, sometimes with poor conditions and incentive, because for some children work is the only means for survival. Therefore, it is important to supplement the social normative approach of eliminating child labor with an economic approach that provide incentives to poor families for them to develop alternative livelihoods.

Indeed, the acceptance of certain occupations, such as farming and fishing as family works, provide support for child labor practices. The normative change program should include measures that train communities to accept the unique career path of every child and the negative implications of forcing children into a supposed family occupation. Education should be acknowledged as the foundation not only for career in the formal sector but also relevant for those working in the informal sector. Technical and vocational education should be promoted as suitable alternatives for children whose parents deem them unfit for theory-oriented educational programs. Studies that seek to further explore the negative social norms and enable community members to provide alternative normative paths are desired. Intervention studies to change the negative social norms and model positive social norms is needed. A holistic assessment of the key promulgation in the UNCRC and ILO’s child labor standards in developing countries is needed to guide amendments and operationalizations within the local context.

### 4.4. Limitations

This study is not without limitations. Our search is limited to only English published academic articles on the subject. It is possible that relevant information on the social norms and child labor may be available in gray literature, including book chapters and reports. Even though we included the two big databases in the social sciences literature; Scopus and Web of Science, studies contained exclusively in other databases may be overlooked. We admit that some studies may have discussed the social norms in child labor without a strict use of the lingua or terminologies of norms, values, beliefs, culture, etc., and thus may have been mistakenly excluded from our review. The documented procedure will allow an updated review to be conducted. Throughout the manuscript we have adopted a loose definition of social norms “rules that is understood and shared by members of the social group (e.g., community) and guide behavior and decisions”. Our intention was to provide non-abstract level interpretations of the various means social norms that inform decisions including child labor. This approach and the way we have sought to argue about social normative interpretations vary slightly from the typical methods a classical cultural or anthropology researcher would use. This does not obliterate our utmost intention of this study, which is tied to practice. Furthermore, there was no pre-published protocol for this review.

## 5. Conclusions

This systematic review advances knowledge on social norms that influence decisions on child labor practices in the family. Findings from the included studies confirmed Parsons’ argument that uncritical acceptance of values as given could be detrimental to society. The findings showed that even though values of respect, hard work and inheritance are important, normative interpretations of them have tendencies of influencing child labor practices within the family. Norms on gender, inheritance and sustenance, value of education and the asset value of children are influential in parents’ decisions to involve their children in work/hard labor. The study findings provide directions for normative change programs targeting the negative interpretations of these values and social norms.

## Figures and Tables

**Table 1:** Boolean search strategy and word combinations.

Keywords and Word Combinations
Child labor * AND culture OR social norm Child labor * AND legislation OR policy
Child work * AND culture OR norm AND Child work AND policy OR law
Child labor AND social standard OR culture AND Working children AND culture OR socialization
Hazardous work OR Labor AND culture AND Worst Form of Child Labor AND child right

Note: (*) is used to identify different ways the word maybe written.

**Table 2:** Summary of studies included in the review.

Reference	Study Method	Sample	Location	Child Labor Activity Explored	Study Purpose	Key Findings
Adonteng-Kissi [61]	In-depth interviews, focus group interviews and observation	60 parents	Ghana	Farming and Fishing	To explore parents’ perception on the nature of child labor	(A) Children engage in child labor in order to take over from their parents’ and the family’s business (B) Children are required to engage in family business as a form of preparation for marriage and future career (C) Without children working family business could collapse
Adonteng-Kissi [62]	In-depth interviews, focus group interviews and observation	60 participants including parents and social service workers	Ghana	Farming and Fishing	Explore the cultural challenges that inhibit the implementation of UNCRC’s provision of children’s right to provision, protection and participation in child labor	(A) Children are socialized to believe in child labor. (B) Parents saw rights as obstruction to socio-cultural child rearing practices (C) Legislation on children’s right were perceived as initiating western parenting styles on Africa.
Adonteng-Kissi [64]	In-depth interviews, and focus group interviews	60 parents, government workers, and workers of NGOs	Ghana	Fishing and Farming	Explore the causes of child labor in Ghana	(A) Children enhance competency and obtain farm ethics through working on family farms (B) Failure to work on family farms is a sign of disrespect (C) Children who engage in family farms secure better future.
Akilova [67]	In-depth narrative interviews	29 participants (12 parents and 17 children)	Tajikistan	General child labor and child work	Explore the pathways to child work and child labor in Tajikistan	(A) Child work is normalized in post-soviet Tajikistan (B) Children are required to work and contribute to their family income.
Bahar [68]	In-depth interviews, demographic survey and observation	27 low-income mothers	Turkey	General child labor	Explore mother’s beliefs and attitudes about child labor in Turkey	(A) Quest to teach children about work and life influenced parents to involve their children in work. (B) Child labor prevent children from the dangers associated with inner cities
Baker & Hilton [69]	Ethnography	162 children and youth	Nepal	Carpet industry	To explore western interpretation of children’s rights and its influence on child labor practices in Nepal	The success of child right promotion programs depends on socio-cultural interpretations.
Adonteng-Kissi [66]	In-depth interviews, focus group interviews and survey	400 survey and 60 parents.	Ghana	Fishing and Farming	To ascertain the impact of child labor policies on child labor in Ghana	(A) Legislations on child labor have had some impact on the rate of child labor, but not severe. (B) Cultural beliefs impede the implementation of child labor legislation.
Berlan [70]	Ethnography and child-friendly participatory method	84 children	Ghana	Cocoa	Explore the impact of micro-level factors on child labor in Ghana	Beliefs over the formative value of work-informed influenced child labor in cocoa.
Bray [71]	Mixed method: Survey and ethnography	5000 surveys in Cape Town and ethnographic engagement in Cape Peninsular	South Africa	Child domestic work	Explore children’s involvement in household work as a form of child labor.	(A) Child domestic activities is gendered with girls undertaking more maintenance roles. (B) Children are contracted by neighbors to engage in paid domestic work. (C) Children cannot refuse instructions from parents and elders to undertake hard work due to the cultural expectation of respect for elders.
Adonteng-Kissi [63]	In-depth individual and focus group interviews	60 parents and social service workers	Ghana	Fishing and Farming	Explore whether child labor violate human right.	(A) Parents consider child labor as part of the best interest of children (B) Children will become wayward if they are granted powers of decision making
Busquet et al. [65]	In-depth individual and focus group interviews	38 key informant interviews and 12 focus groups interviews	Ghana and Cote d’Ivoire	Cocoa	Explore the value chain processes in child labor within cocoa areas	(A) Child labor is enforced by the normative belief that children should follow the path of their parents, and children are required to undertake informal apprenticeship training to succeed in life.
Delap [72]	In-depth interviews	45 participants	Bangladesh	General child labor	Explore economic and cultural factors that underpin child labor	The type of work children will do at home or in the carpet industry is determined by gender roles.
Krauss [73]	Mixed method: Survey and Interviews	8687 household survey in the GLSS data, and 15 qualitative interviews with children and the Minister of Education	Ghana	General child labor	Explore monetary and non-monetary factors that influence child labor in sub-Saharan Africa	Structure of the economy and social norm of inheriting farming occupation are the main driving forces of child labor in Africa.

Note: GLSS = Ghana Living Standards Survey.

## Data Availability

Not applicable.
